# Essential Oils from *Zingiber striolatum* Diels Attenuate Inflammatory Response and Oxidative Stress through Regulation of MAPK and NF-κB Signaling Pathways

**DOI:** 10.3390/antiox10122019

**Published:** 2021-12-19

**Authors:** Zebin Huang, Lingna Xie, Yongyu Xu, Kai Zhao, Xuetong Li, Jiaben Zhong, Yujing Lu, Xuetao Xu, Susan Goodin, Kun Zhang, Lanyue Zhang, Chunlian Li, Xi Zheng

**Affiliations:** 1 School of Biomedical and Pharmaceutical Sciences, Guangdong University of Technology, Guangzhou 510006, China; 1112012002@mail2.gdut.edu.cn (Z.H.); 1112012007@mail2.gdut.edu.cn (L.X.); 2112012012@mail2.gdut.edu.cn (Y.X.); 2111906138@mail2.gdut.edu.cn (K.Z.); 2112012028@mail2.gdut.edu.cn (X.L.); 2112112008@mail2.gdut.edu.cn (J.Z.); luyj@gdut.edu.cn (Y.L.); 2 School of Biotechnology and Health Sciences, Wuyi University, Jiangmen 529020, China; xuetaoxu@wyu.edu.cn (X.X.); kzhang@gdut.edu.cn (K.Z.); 3 Rutgers Cancer Institute of New Jersey, The State University of New Jersey, New Brunswick, NJ 08903, USA; goodin@cinj.rutgers.edu; 4 Guangdong Provincial Key Laboratory of Plant Resources Biorefinery, Guangdong University of Technology, Guangzhou 510006, China; 5 Susan Lehman Cullman Laboratory for Cancer Research, Department of Chemical Biology, Ernest Mario School of Pharmacy, Rutgers, The State University of New Jersey, Piscataway, NJ 08854, USA

**Keywords:** *Zingiber striolatum* Diels, essential oil, oxidant stress, anti-inflammatory activity, MAPK, NF-κB

## Abstract

*Zingiber striolatum* Diels (*Z. striolatum*), a widely popular vegetable in China, is famous for its medicinal and nutritional values. However, the anti-inflammatory effects of essential oil from *Z. striolatum* (EOZS) remain unclear. In this study, EOZS from seven regions in China were extracted and analyzed by GC–MS. LPS-induced RAW264.7 cells and 12-*O*-Tetradecanoylphorbol 13-acetate (TPA)-stimulated mice were used to evaluate the anti-inflammatory effects of EOZS. Results show that 116 compounds were identified in EOZS from seven locations. Samples 2, 4 and 5 showed the best capability on DPPH radical scavenging and NO inhibition. They also significantly reduced the production of ROS, pro-inflammatory cytokines, macrophage morphological changes, migration and phagocytic capability. Transcriptomics revealed MAPK and NF-κB signaling pathways may be involved in the anti-inflammatory mechanism, and the predictions were proven by Western blotting. In TPA-induced mice, EOZS reduced the degree of ear swelling and local immune cell infiltration by blocking the activation of MAPK and NF-κB signaling pathways, which was consistent with the in vitro experimental results. Our research unveils the antioxidant capability and potential molecular mechanism of EOZS in regulating inflammatory response, and suggests the application of EOZS as a natural antioxidant and anti-inflammatory agent in the pharmaceutical and functional food industries.

## 1. Introduction

Inflammation, the basis of many kinds of pathological processes, is an automatic defense response of the human body. It is generally triggered by infection, tissue injury, tissue stress and malfunction [1]. The primary role of inflammation is to eliminate harmful stimuli, prevent further damage, start the healing process and recover the normal functions of injured tissues [2]. However, host tissue homeostasis will be disarranged or damaged when inflammation is excessive or uncontrolled [3], which may induce diseases such as acute cytokine storm [4], rheumatoid arthritis [5], atherosclerosis [6] and cancer. In dysregulated inflammatory response, macrophages are hyperactivated to induce oxidant stress and produce more inflammatory media such as reactive oxygen species (ROS), nitric oxide (NO), Prostaglandin E2 (PGE_2_), Interleukin-6 (IL-6), Interleukin-1β (IL-1β) and tumor necrosis factor alpha (TNF-α), thereby further aggravating the progression of inflammation [7,8]. The imbalance of oxidative stress caused by inflammation may also lead to disease exacerbations [9]. For example, the severity of COVID-19 patients is affected by oxidative stress [10]. Excessive NO and ROS will destroy lipids, proteins and DNA in cells, and damage the normal function of tissues [11,12]. In order to reduce the risk of inflammation-related diseases, it is of great significance to suppress dysregulated inflammatory response. Thus, the development of anti-inflammatory agents is still a common concern of many scientists. 

In recent years, people have become more aware of the health benefits of natural products derived from fruits, vegetables or other plants, and have shown great interest in developing natural anti-inflammatory agents. Essential oil is a kind of volatile aromatic substance extracted from plants. As a kind of traditional medicine, essential oils have attracted widespread attention because of their potential pharmacological activities including anti-inflammatory, antioxidant [13], antibacterial [14] and anticancer potential [15], etc. 

*Zingiber striolatum* Diels, commonly known as Yang-He in Chinese, belongs to *Zingiber* species. As a unique vegetable grown in China, the edible part of *Z. striolatum* is the flower. It is widely distributed and predominantly wild but less cultivated [16]. *Z. striolatum* possesses high nutritional value because it contains a variety of amino acids, proteins and rich cellulose [17]. The total amino acid content in *Z. striolatum* is 19.83%, of which essential amino acids account for 37.17% of the total amino acids [18]. A recent study has suggested that the ethanol extract from *Z. striolatum* exerted noticeable hypoglycemic activity in vitro [17]. Tian et al. revealed that the essential oil of *Z. striolatum* from the rhizome has weak antioxidant activity but strong antimicrobial and anticancer activities [19]. They further found that essential oils of *Z. striolatum* from flowers, leaves and stems showed significant cytotoxicity against K562, PC-3 and A549 cells [20]. According to the Compendium of Materia Medica, a famous Chinese medicine classic compiled by Shizhen Li during the 16th century, *Z. striolatum* was used for promoting blood circulation, eliminating phlegm, alleviating coughing and relieving swelling and pain, which means it has potential anti-inflammatory activity. However, the regulatory effect of *Z. striolatum* on inflammatory response and its molecular mechanism remain to be elucidated. Therefore, the aim of our study was to assess the anti-inflammatory activity of EOZS and explore its potential molecular mechanism in vitro and in vivo. To understand the material basis of EOZS, we also analyzed their chemical composition from different districts in China by GC–MS.

## 2. Materials and Methods

### 2.1. Plant Materials and Extraction of EOZS

*Z. striolatum* were collected from seven different locations, shown in Table S1. The fresh flowers were harvested from April to August. All plant materials were identified with the kind assistance of Professor Nian Liu, a senior botanist from Zhongkai University of Agriculture and Engineering.

The flowers were air-dried under normal temperature and then crushed. The powder (1 kg) was mixed with deionized water (5 L) in a 10 L round bottomed flask for 6 h steam distillation. The volatile components are condensed through a condenser tube and collected into a conical flask. Then, the essential oils were dried with anhydrous sodium sulfate and preserved in tubes at 4 °C for further study.

### 2.2. Chemicals and Reagents

Lipopolysaccharide (LPS), 2,2-Diphenyl-1-picrylhydrazyl (DPPH), 12-*O*-Tetradecanoylphorbol 13-acetate (TPA), Thiazolyl blue tetrazolium bromide (MTT) and 2′,7′-Dichlorodihydrofluorescein diacetate (DCFH-DA) were purchased from Sigma-Aldrich (St. Louis, MO, USA). 4′,6-Diamidino-2-phenylindole dihydrochloride (DAPI) was purchased from Beijing Solarbio Science and Technology Co., Ltd. (Shanghai, China). N-Alkane Solution (C_7_ to C_40_, 1000 mg/L) was obtained from O2Si, LGC Standards (Charleston, SC, USA). Dulbecco’s modified Eagle’s medium (DMEM), fetal bovine serum (FBS) and 100 U/mL penicillin and 100 mg/L streptomycin were purchased from Gibco, Thermo Fisher Scientific (Waltham, MA, USA). NO assay kit, COX2 antibody and secondary anti-rabbit antibodies were bought from Beyotime Biotechnology Co., Ltd. (Shanghai, China). IL-6, TNF-α and Prostaglandin E_2_ (PGE_2_) ELISA kit were provided by Jiangsu Meibiao Biological Technology Co., Ltd. (Jiangsu, China). INOS antibodies were acquired from Affinity Biosciences (Cincinnati, OH, USA). P38, phosphor-p38, ERK1/2, phosphor-ERK1/2, SAPK/JNK, phosphor-SAPK/JNK, p65, Lamin B1, β-actin antibodies and secondary anti-rabbit IgG antibodies conjugated with Alexa Fluor 555 were purchased from Cell Signaling Technology (Danvers, MA, USA). FITC-dextran (4 kDa) and other reagents of analytical grade were purchased from Aladdin (Shanghai, China).

### 2.3. Analysis of Chemical Compounds of EOZS

The chemical compounds of EOZS were analyzed by a Shimadzu instrument (GCMS-QP2010 Ultra), equipped with a Rxi-5 ms silica capillary column (30 m × 0.25 mm, 0.25 μm film thickness). The operating method was performed according to a previous study [21] with few modifications. The temperature of the injector was set to 250 °C. The oven’s initial temperature was set to 60 °C, and raised to 195 °C at a rate of 3 °C/min. Then, the temperature was increased to 300 °C at 15 °C/min and held at this temperature for 10 min. Helium was used as a carrier gas and its linear velocity was adjusted to 36.5 cm/s (1.0 mL/min). Injected volumes of the sample were 1 μL in split mode (oil 50 μL: hexane 500 μL). The ionization energy was set to 70 eV. The transfer line temperatures and ionization source were kept at 250 °C and 200 °C, respectively. Mass spectra were obtained in the range of 35–400 *m*/*z*, with a scanning speed of 0.3 s/scan. The retention index of each compound was calculated by using n-alkanes standards (C_8_–C_40_). The compounds were identified by comparing the mass spectra of each chromatographic peak in the NIST05 library and comparing their calculated retention indexes with the retention indexes in the literature [22,23,24,25,26,27,28,29,30,31,32,33,34,35,36,37].

### 2.4. Antioxidant Assay

The DPPH radical scavenging assay was used to evaluate the antioxidant capacity of EOZS. In short, 0.1 mM DPPH (dissolved in methanol) was added to a 96-well plate in a volume of 150 μL per well. Subsequently, gradient concentrations of EOZS (80, 40, 20, 10 μg/mL) were added. The 96-well plate was protected from light and incubated for 30 min at room temperature. The absorbance values were measured at 517 nm using a microplate reader. DPPH scavenging capacity was calculated using the following formula, and the results were presented as percentage:

DPPH scavenging capacity (%) = [1 − (OD_sample_/OD_control_)] × 100%

### 2.5. Cell Culture and Detected of Cytotoxicity

RAW 264.7 macrophage cells were purchased from the Shanghai Zhong Qiao Xin Zhou Biotechnology Co.; Ltd (Shanghai, China). The cells were maintained in DMEM/high-glucose containing 10% FBS, 100 U/mL penicillin and 100 mg/L streptomycin. Cells were grown in a humidified atmosphere supplemented with 5% CO_2_ at 37 °C. The cytotoxicity of EOZS on RAW 264.7 cells were determined by MTT. In brief, RAW 264.7 cells (5 × 10^4^ cells/mL) were plated in a 96-well plate and maintained for 24 h. Then, the cells were treated with EOZS at various concentrations (0, 5, 10, 20, 40, 80 μg/mL) and incubated for another 24 h. After removing the spent medium, 100 μL MTT solutions (dissolved in serum-free DMEM, 500 μg/mL) were added to each well and cultured for 4 h. Then, the supernatant was discarded and formazan crystals were dissolved in DMSO (150 μL/well). Finally, the absorbance values were detected at 570 nm. Data were presented in the form of percentages.

### 2.6. Determination of NO Production

RAW 264.7 cells (5 × 10^5^ cells/mL) were seeded in a 24-well plate and incubated overnight. After discarding the supernatant, the cells were pre-treated with EOZS at different concentrations for 2 h, and then incubated with LPS (1 μg/mL) for 24 h [38]. NO production was measured using a NO assay kit according to the specification. In short, 50 μL supernatant of each well was transferred to another 96-well plate, followed by mixing with an equal volume of Griess reagents for 10 min. The absorbance values at 540 nm were determined using a microplate reader. The concentrations of NO were calculated according to the standard curve of NaNO_2_.

### 2.7. Determination of Intracellular ROS 

Intracellular ROS produced by LPS-induced RAW 264.7 cells were detected using the DCFH-DA fluorescent probe, which can be oxidized by ROS in cells to produce green fluorescent DCF. Thus, the level of intracellular ROS is proportional to the fluorescence intensity of DCF. Briefly, RAW 264.7 cells were seeded in 6-well plate at a density of 1 × 10^6^ cells per well and maintained for 24 h. The cells were treated with 40 μg/mL of S2, S4 and S5 for 2 h, and were co-treated with LPS (1 μg/mL) for an additional 24 h. Then, the cells were incubated with DCFH-DA for 30 min and subsequently washed with PBS for three times. After blowing down the cells, the cells were resuspended with PBS and 10 μL was taken in a counting plate and placed in Countess™ II FL Automated Cell Counter (ThermoFisher, Waltham, MA, USA) for counting and photographing.

### 2.8. ELISA Assay

The level of pro-inflammatory cytokines (TNF-α, IL-6 and PGE_2_) was determined using ELISA kits according to the manufacturer’s instructions. In brief, 50 μL of standards, samples or blank were added to the wells pre-coated with antibodies, and incubated at 37 °C for 30 min followed by washing each well five times with 200 μL of wash solution. Then, 50 μL of HRP-conjugate reagent with antibodies was added to each well and incubated at 37 °C for 30 min. After a washing step with 200 μL of wash solution for five times, 100 μL of TMB substrate solution was added and incubated at 37 °C for 10 min. Then, 50 μL of stop solution was added to each well to stop the reaction. The absorbance values were obtained at 450 nm. The concentrations of cytokines were calculated according to the relevant standard curve.

### 2.9. Analysis of Cell Morphology and Size

RAW 264.7 cells (5 × 10^5^ cells/mL) were plated in a 6-well plate and incubated overnight. After pre-treatment with 40 μg/mL of S2, S4 and S5 for 2 h, the cells were co-treated with LPS (1 μg/mL) for another 12 h and monitored with a HoloMonitor M4 instrument (PHI, Lund, Sweden). Time-lapse images of each well were captured from three random positions. Data on the optical volume, area and average optical thickness of RAW 264.7 cells were obtained from HoloStudio M4 software. 

### 2.10. Cell Migration Assay

An in vitro scratch assay was used to assess the ability of cell migration. Briefly, RAW 264.7 cells were seeded in a 12-well plate and incubated overnight to form over a 90% confluent monolayer. Then, cells were pre-treated with indicated EOZS in 1% FBS medium for 2 h, followed by scratching using a pipette tip and co-treatment with LPS (1 μg/mL) for 24 h. Images were captured at 0 h and 24 h, and cell migration distance was measured by using image J (NIH Image J system, Bethesda, MD, USA).

### 2.11. Determination of Phagocytosis

Phagocytic uptake of RAW 264.7 cells was detected by using FITC-dextran. RAW 264.7 cells (1 × 10^6^ cells per well) were seeded in a 6-well plate and incubated for 24 h. Cells were then treated with S2, S4 and S5 for 2 h, followed by co-treatment with LPS (1 μg/mL) for 24 h. After discarding the supernatant, cells were incubated with fresh medium containing FITC-dextran (1 mg/mL) at 37 °C for 1 h. The phagocytosis of cells on FITC-dextran were analyzed by Flowsight flow cytometry (Merck, Schwalbach, Germany).

### 2.12. RNA Sequencing

Three groups (three samples per group) were used for the analysis of RNA-seq, including control, LPS, LPS + S4 (briefly indicated as S4). Total RNA from three groups were obtained using Trizol reagent (Invitrogen, Carlsbad, CA, USA). After the RNA purity, concentration and integrity of each sample were tested for eligibility, cDNA libraries were constructed and sequenced using the Illumina Novaseq platform by PE150 strategy.

### 2.13. Western Blotting 

RIPA lysis buffer containing Protease and Phosphatase Inhibitor Cocktail (Beyotime Technology, Shanghai, China) was added to RAW 264.7 cells and then incubated in ice for 10 min, followed by centrifugation at 12,000 rpm for 10 min at 4 °C. After collecting the supernatant, the total protein concentration of each sample were determined using a BCA Protein Assay Kit (Beyotime Technology, Shanghai, China). Specifically, the protein of cell nucleus and cytoplasm were separated, respectively, using a NE-PER Nuclear and Cytoplasmic Extraction Reagents (Thermo Scientific, Waltham, MA, USA). The protein samples were mixed with loading buffer and boiled at 100 °C for 10 min to denature. Subsequently, the proteins were separated by 8% SDS-PAGE gels, transferred to PVDF membranes (Millipore, Burlington, MA, USA) and blocked with 5% BSA (Aladdin, Shanghai, China). The membranes were incubated with the primary antibodies (1:1000) diluted in Primary Antibody Dilution Buffer (Beyotime Technology, Shanghai, China) at 4 °C overnight, and then incubated with secondary antibodies (1:2000) diluted in TBST for 2 h. At last, the proteins were determined using Immobilon Western Chemilum HRP Substrate (Millipore, USA).

### 2.14. Immunofluorescence Staining

RAW 264.7 cells were seeded in 35 mm confocal dishes at a density of 5 × 10^5^ cells/ dish and maintained for 24 h. After treating with S2, S4 and S5 (40 μg/mL) for 2 h, the cells were co-incubated with LPS (1 μg/mL) for another 24 h. After fixing with 4% paraformaldehyde solution for 10 min, the cells were permeabilized by 0.5% Triton X-100 solution treatment for 15 min and incubated with primary antibody (p65, 1:400) at 4 °C overnight. Then, the cells were incubated with secondary antibody conjugated with Alexa Fluor 555 for 2 h at room temperature, followed by treatment with DAPI solution (10 μg/mL) for 5 min. Image acquisitions were performed using Zeiss LSM-800 scanning confocal microscope (Jena, Germany).

### 2.15. TPA-Induced Mouse Ear Edema Model

A total of twenty-nine male Kunming (6-week-old) mice were purchased from Guangdong Medical Laboratory Animal Center (Guangzhou, China). Mice were acclimatized for seven days under temperature (25 °C) and relative humidity (50%) controlled conditions and a 12 h light/dark cycle before the experiments. Subsequently, all the mice were randomly divided into six groups, including control (*n* = 4), TPA (*n* = 5), dexamethasone (DXM, *n* = 4), S2 (*n* = 6), S4 (*n* = 5) and S5 (*n* = 5). DXM (5 mg/kg), S2 (50 mg/kg), S4 (50 mg/kg) and S5 (50 mg/kg) were, respectively, administrated to the right ear of mice for two days. On the third day, one hour after administration with DXM, S2, S4 and S5, 20 μL of TPA (50 μg/mL) was applied to the right ear of mice, except for the control group. After six hours, mice were euthanized and the right ear tissues (6 mm diameter) obtained from each mouse were weighed. All procedures in the experiment were performed according to the national legislation and local guidelines of Wuyi University (Approval number: CN2021001).

### 2.16. Hematoxylin–Eosin Staining

Mice ear tissues were fixed with 4% paraformaldehyde, embedded with paraffin, and made into sections. The tissue sections were stained using hematoxylin–eosin (HE) observed and photographed using Olympus IX71 microscope (Tokyo, Japan). 

### 2.17. Immunohistochemistry Staining

After dewaxing and rehydrating with a gradient of ethanol and distilled water, the sections were doused with 3% H_2_O_2_ for 30 min. Then, the sections were blocked with 10% goat serum for 30 min, incubated with primary antibodies against p-p65, p-p38, p-ERK1/2, p-JNK1/2 at 4 °C overnight and then incubated with secondary antibody. Finally, the sections were stained with 3,3′-diaminobenzidine and photographed using Olympus IX71 microscope.

### 2.18. Statistical Analysis

PAST (Version 3.0) software was used to perform matrix plot, principle component analysis (PCA) and hierarchical cluster analysis (HCA) on 116 chemical components in EOZS from seven regions. Grayscale values of bands in Western blotting were analyzed using Image J (Rockville, MD, USA). The integral optical density (IOD) of immunohistochemical staining was analyzed using Image Pro Plus (Rockville, MD, USA). All the data in this study were presented as the mean ± standard error of mean (SEM). Statistical significance was assessed by one-way analysis of variance (ANOVA) using GraphPad Prism 7.0 software. A value of *p* < 0.05 was considered to represent a significant difference.

## 3. Results

### 3.1. The Compositions, PCA and HCA of EOZS from Different Regions

**Figure 1:**
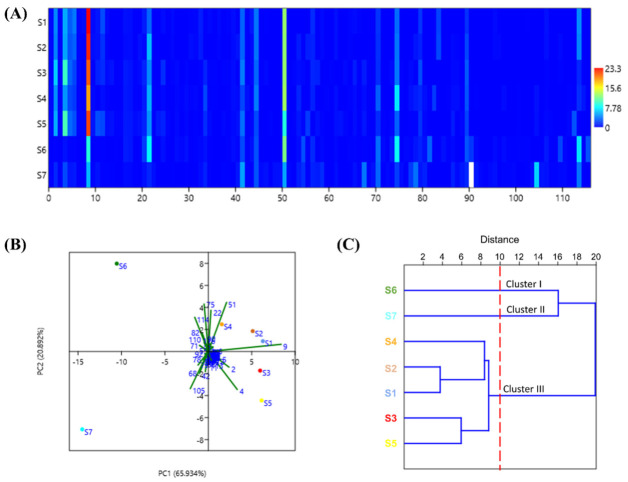
Matrix plot, PCA and HCA of EOZS from different locations: (**A**) matrix plot obtained by relative content of chemical compounds from S1 to S7; (**B**) PCA obtained by EOZS from seven locations; and (**C**) HCA of EOZS from different locations based on UPGMA method.

Samples of *Z. striolatum* were collected from seven cities (Table S1) in China and their essential oil compositions were analyzed using GC–MS. The results show that a total of 116 chemical compounds were identified from seven samples (Table 1). In order to visually observe the relative content differences of EOZS from different locations, we generated a matrix plot based on the variables of 7 samples × 116 components (Figure 1A). Each color unit corresponds to the relative content of each compound, and from red to blue represents the relative content from high to low. Obviously, there were differences in the components and relative contents of EOZS from different locations. The main components and contents of S1~S5 were similar, including β-phellandrene (18.38~23.34%), α-humulene (8.77~12.38%), β-pinene (4.53~10.01%), α-pinene (3.93–6.26%), cryptone (3.08–5.74%), and 2,5,9-trimethylcycloundeca-4,8-dienone,8-dienone (2.95–7.12%). The main components of S6 were α-humulene (11.58%), β-phellandrene (8.81%), 2,5,9-trimethylcycloundeca-4,8-dienone (8.09%), cryptone (7.57%) and tetracosane (6.94%), while the main components of S7 were heptadecanoic acid (6.51%), β-phellandrene (5.60%), α-humulene (5.03%) and β-elemene (4.97%). Sixteen common chemical constituents were present in EOZS from the seven locations, namely α-pinene, β-pinene, β-myrcene, α-phellandrene, β-phellandrene, γ-terpinene, cis-p-menth-2-en-1-ol, trans-p-menth-2-en-1-ol, (−)-terpinen-4-ol, cryptone, β-elemene, β-caryophyllene, α-humulene, caryophyllene oxide, 2,5,9-trimethylcycloundeca-4,8-dienone and α-cadinol. In addition, the total percentage of identified compounds in S1–S6 exceeded 80%, while S7 was relatively low at 71.76%.

Principle component analysis is one of the multivariate statistical methods used to identify the most significant features in a data set. PCA pattern recognition of EOZS from different locations can be used to evaluate the compositional differences caused by inter-species or intra-species variation. The cumulative contribution rate of variance of the first two principal components (PC1 and PC2) extracted from the PCA method was 86.82%, which can explain most of the variance information. Therefore, PC1 and PC2 were selected to determine the phytochemical differences of EOZS from different locations. As shown in Figure 1B, the variance contribution rate of PC1 was 65.88%, which was positively correlated with β-phellandrene (9), β-pinene (4) and α-pinene (2). The variance contribution rate of PC2 was 20.94%, of which α-humulene (51), cryptone (22) and 2,5,9-trimethylcycloundeca-4,8-dienone (75) have higher positive contributions. In addition, hierarchical cluster analysis showed the phytochemical distances between EOZS from different locations, which can reflect the relationship between them. *Z. striolatum* from different locations can be divided into three clusters (Figure 1C). S1~S5, characterized by a relatively high content of β-phellandrene, were classified as a cluster. S6 and S7 were in different two clusters, which were characterized by the relatively high content of α-humulene and heptadecanoic acid, respectively. In general, the results of PCA and HCA provide a reference for the quality assessment of EOZS from different locations.

### 3.2. Evaluation of the Antioxidant Activity of EOZS

#### 3.2.1. DPPH Scavenging Activity

**Figure 2:**
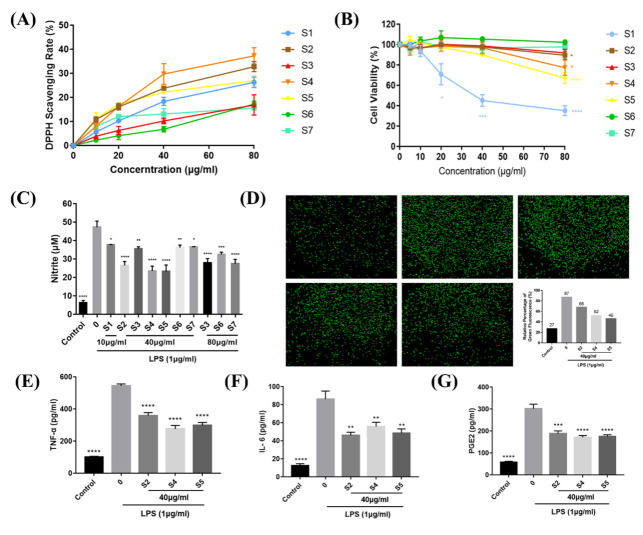
Effects of EOZS on DPPH scavenging and pro-inflammatory mediators released by LPS-induced RAW 264.7 cells: (**A**) DPPH scavenging rate of EOZS from seven locations; (**B**) survival rate of RAW 264.7 cells incubated with EOZS (5, 10, 20, 40, 80 μg/mL) for 24 h; (**C**) NO production in supernate of RAW 264.7 cells pretreated with different concentrations of EOZS for 2 h, followed by co-treatment with LPS (1 μg/mL) for 24 h; (**D**) ROS; (**E**) TNF-α; (**F**) IL-6; and (**G**) PGE_2_ level in RAW 264.7 cells pretreated with 40 μg/mL of EOZS (S2, S4 and S5) for 2 h, followed by co-treated with LPS (1 μg/mL) for 24 h. Values are expressed as means ± SEM (*n* = 3). * *p* < 0.05, ** *p* < 0.01, *** *p* < 0.001, **** *p* < 0.0001, compared with (**B**) untreated group or only (**C**,**E**–**G**) LPS treatment group.

As shown in Figure 2A, EOZS from seven regions all exhibited concentration-dependent antioxidant capacity ranging from 10 μg/mL to 80 μg/mL. At a concentration of 80 μg/mL, the DPPH scavenging rate of EOZS followed the order of S4 (37.26%) > S2 (32.79%) > S5 (26.99%) > S1 (26.26%) > S6 (17.23%) > S3 (16.86%) > S7 (15.61%). 

#### 3.2.2. Effects of EOZS on NO and ROS in LPS-Induced RAW 264.7 Cells

EOZS from seven locations showed different cytotoxicity on RAW 264.7 cells when treated at the same concentration (Figure 2B). The toxicity of S1 on RAW 264.7 cells showed a concentration dependence, with significant effects occurring at concentrations greater than or equal to 20 μg/mL. However, S3, S6 and S7 exhibited no inhibition on the cell proliferation of RAW 264.7 cells at a concentration of 80 μg/mL. The safe concentration of S2, S4 and S5 on RAW 264.7 cells were thought to be 40 μg/mL. Therefore, 10 μg/mL (S1), 40 μg/mL (S2 to S7) and 80 μg/mL (S3, S6 and S7) of different EOZS were selected to clarify their effects on NO release in LPS-induced RAW 264.7 cells. As shown in Figure 2C, NO content in the LPS-treated group was elevated approximately 7.6-fold compared to the control group. After treatment with 10 μg/mL of S1, 40 μg/mL of S2 to S7 and 80 μg/mL of S3, S6, and S7, the mean NO release level of RAW 264.7 cells was decreased by 1.5~3.9-fold. In general, the EOZS from seven regions all significantly inhibited the NO produced by LPS-induced RAW 264.7 cells. In subsequent experiments, EOZS from three different regions with the best effect on NO inhibition (40 μg/mL of S2, S4, and S5) were selected to further investigate their anti-inflammatory effects and underlying molecular mechanism.

ROS, as a second messenger in the inflammatory response, is upregulated during inflammation, thereby inducing oxidative stress. As shown in Figure 2D, the expression of ROS in RAW 264.7 cells was extremely low in the control group. After treatment with LPS, the cellular ROS level was significantly increased, whereas the level of ROS expression in RAW 264.7 cells decreased with the treatment of S2, S4 and S5 at a concentration of 40 μg/mL.

### 3.3. EOZS Attenuated Production of Pro-Inflammatory Mediators

To further investigate the inhibitory effect of EOZS on inflammatory factors, we collected the supernatants of LPS-induced RAW 264.7 cells for detection. Compared with the control group, the expression levels of TNF-α, IL-6 and PGE_2_ in LPS-induced RAW 264.7 cells were significantly increased (*p* < 0.0001), up-regulated by 5.40-, 6.85- and 5.24-fold, respectively. Treatment with 40 μg/mL of S2, S4 and S5 caused a significant decrease in TNF-α, IL-6 and PGE_2_ production (Figure 2E–G). 

### 3.4. EOZS Reduced Cellular Morphological Changes, Migration and Phagocytic Capability in LPS-Induced RAW 264.7 Cells

**Figure 3:**
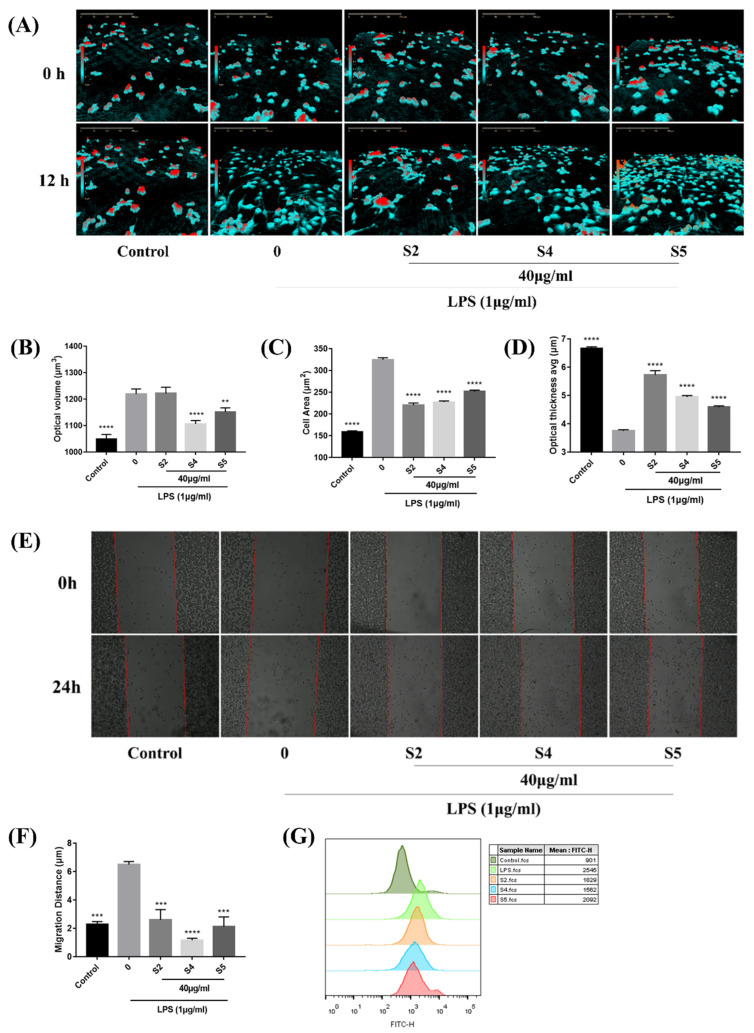
Effects of EOZS on cell morphological changes, migration and phagocytic capability: (**A**) cell morphology; (**B**) optical volume; (**C**) cell area; and (**D**) optical thickness average of RAW 264.7 cells pretreated with 40 μg/mL of EOZS (S2, S4 and S5) for 2 h, followed by co-treatment with LPS (1 μg/mL) for 12 h; (**E**) Images of cell migration and (**F**) quantified migratory distance in LPS-induced RAW 264.7 cells treated with an indicated concentration of EOZS; (**G**) phagocytosis of FITC-dextran in LPS-induced RAW 264.7 cells treated without or with EOZS were analyzed by flow cytometry. Values are expressed as means ± SEM (*n* = 3). ** *p* < 0.01, *** *p* < 0.001, **** *p* < 0.0001, compared with only LPS treatment group.

During the inflammatory process, activated macrophages alter their morphology and phenotype to rapidly respond to external stimuli. Several studies have shown that cell morphology, migration and phagocytic capability would be changed in activated RAW 264.7 cells [39,40,41]. It could be distinctly observed that 12 h after LPS stimulation, RAW 264.7 cells underwent deformation and produced elongated pseudopods, accompanied by an increase in cell volume and area as well as a decrease in cell thickness, while these conditions were improved in the S2, S4 and S5 treatment group (Figure 3A–D). Furthermore, S2, S4 and S5 significantly inhibited the migration ability in LPS-stimulated RAW 264.7 cells (Figure 3E,F). We also tested the effect of S2, S4 and S5 on the phagocytic activities of LPS-induced RAW 264.7 cells by using FITC-labeled dextran. Flow cytometry data showed that all three reduced the phagocytic ability in LPS-induced RAW 264.7 cells (Figure 3G).

### 3.5. Bioinformatics Analysis of RNA Sequencing Data

**Figure 4:**
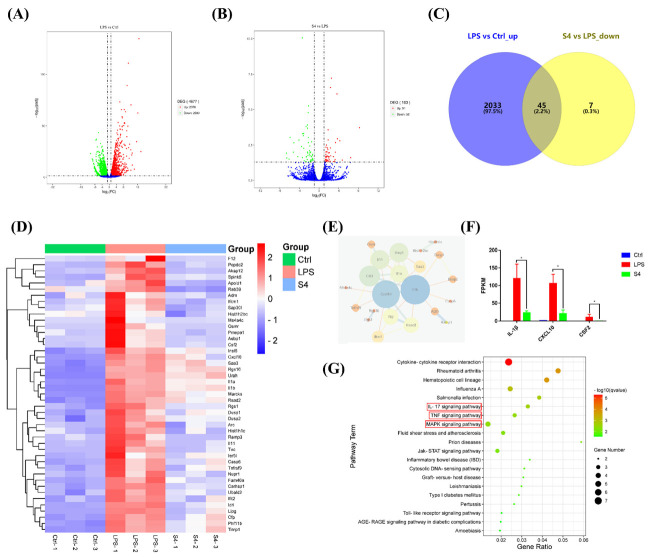
Effects of EOZS on the transcription of LPS-induced RAW 264.7 cells. Volcano plot shows DEGs in (**A**) the LPS vs. control groups; and (**B**) S4 vs. LPS groups. Forty-five DEGs that were up-regulated by LPS and down-regulated by S4 were (**C**) intersected and exhibited in the (**D**) heatmap. Relationship of 45 co-regulated DEGs were visualized in (**E**) protein–protein interaction network and (**F**) FPKM values of three hub genes were screened; (**G**) KEGG enrichment analysis of 45 co-regulated DEGs. Values are expressed as means ± SEM (*n* = 3). * *p* < 0.05, compared with only LPS treatment group.

To investigate the mechanism of EOZS-inhibiting LPS-induced RAW 264.7 cells, control, LPS and S4 groups were selected for RNA sequencing. Gene expression correlation analysis suggested Pearson correlation coefficient between different samples in the same group were between 0.973 and 0.998, indicating the excellent biological replicates among the samples (Figure S1A). Compared with the control group, 4677 differentially expressed genes (DEGs) were identified in LPS-induced RAW264.7 cells, of which 2078 were up-regulated and 2599 were down-regulated (Figure 4A). A total of 103 DEGs were found in S4 vs. LPS group, of which 51 were up-regulated and 52 were down-regulated (Figure 4B). Then, we calculated the intersections between 2078 up-regulated DEGs in the LPS vs. control groups and 52 down-regulated DEGs in the S4 vs. LPS group. As shown in the Venn diagram and the heat map, 45 co-regulated DEGs were obtained (Figure 4C,D). String (https://www.string-db.org/, accessed on 21 September 2021) was performed to analyze protein–protein interaction network of 45 co-regulated DEGs (confidence score = 0.7), and disconnected nodes in the network were hided. Three hub genes with the highest degree were visualized using Cytoscape software (Figure 4E), namely IL-1b (12), CXCL10 (11) and CSF2 (7), and their FPKM value in the three groups were presented in Figure 4F.

To gain a better understanding of the potential relationship among the 45 co-regulated DEGs, we conducted a functional enrichment analysis. GO analysis revealed that the biological process of these DEGs included the regulation of nitric-oxide synthase activity, inactivation of MAPK activity, etc. Cellular components were mainly extracellular space and extracellular region, while molecular function involved MAP kinase tyrosine/serine/threonine phosphatase activity, interleukin-1 receptor binding, etc. (Figure S1B). In accordance with the results of KEGG enrichment, S4 treatment regulated IL-17 signaling pathway, TNF signaling pathway and MAPK signaling pathway (Figure 4G). We further found through the KEGG map that MAPK signaling pathway and NF-kB signaling pathway were involved in the IL-17 signaling pathway and TNF signaling pathway (Figure S1D). Based on the evidence above, Western blotting was subsequently performed to verify the inhibitory effect of S4 on the MAPK signaling pathway and NF-kB signaling pathway.

### 3.6. EOZS Inhibited Expression Level of iNOS, COX2 and Activation of MAPK and NF-κB Pathways in LPS-Induced RAW 264.7 Cells

**Figure 5:**
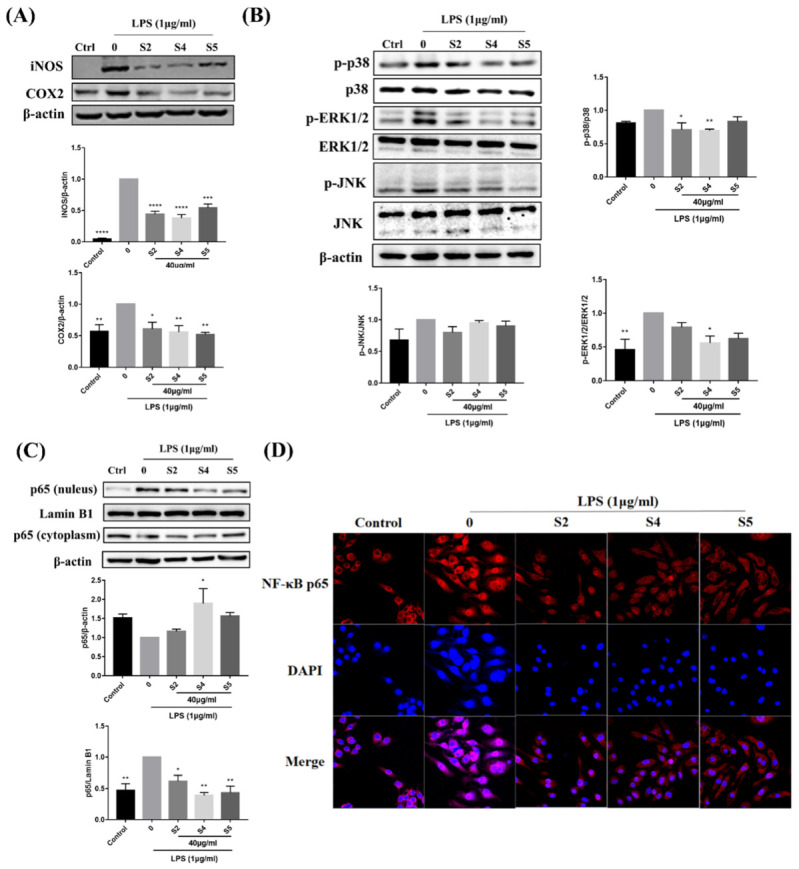
Effects of EOZS on protein levels of the iNOS, COX2, MAPK and NF-κB signaling pathways. RAW 264.7 cells were pretreated with 40 μg/mL of EOZS (S2, S4 and S5) for 2 h, followed by co-treated with LPS (1 μg/mL) for 24 h (**A**,**C**,**D**) or 30 min (**B**). Values are expressed as means ± SEM (*n* = 3). * *p* < 0.05, ** *p* < 0.01, *** *p* < 0.001, **** *p* < 0.0001, compared with only LPS treatment group.

NO and PGE_2_ secreted by the cells can be catalyzed by inducible nitric oxide synthase (iNOS) and Cyclooxygenase 2 (COX2), respectively. Therefore, the above results prompted us to investigate the changes in the protein expression of iNOS and COX2. As expected, the dramatic up-regulation of iNOS and COX2 proteins was observed in the LPS treatment group, while 40 μg/mL of S2, S4 and S5 treatment reduced the expressions of both proteins (Figure 5A).

After LPS treatment for 30 min, the MAPK signaling pathway in LPS-induced RAW 264.7 cells was activated, as evidenced by significantly increased protein phosphorylation levels of p38, ERK1/2 and JNK. Interestingly, 40 μg/mL of S2, S4 and S5 treatment all reduced the expression levels of p-p38, p-ERK1/2 and p-JNK to various extents in LPS-induced RAW 264.7 cells, but did not affect the expression of p38, ERK1/2 and JNK (Figure 5B). 

NF-κB p65 protein in the LPS group was significantly increased in the nucleus and decreased in the cytoplasm, indicating the activation of NF-κB signaling pathway, while S2, S4 and S5 treatment reduced the nuclear translocation of NF-κB p65 (Figure 5C). A similar result was detected in the immunofluorescence assay. As shown in Figure 5D, NF-κB p65 marked with red fluorescence in the LPS group was mainly located in the nucleus compared to the control group. However, this condition was reduced in the S2, S4 and S5 treatment groups, suggesting that the nuclear translocation of p65 was obviously blocked. 

### 3.7. EOZS Ameliorated Ear Edema and Infiltration of Immune Cells in TPA-Induced Mouse Model

**Figure 6:**
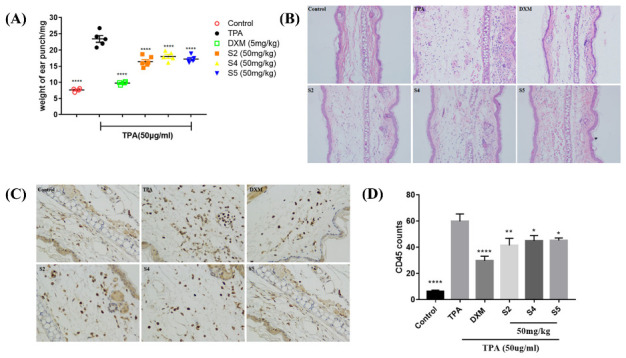
Effects of EOZS on the weight and CD 45 positive cells of mice ear in TPA-induced mice: (**A**) the weight of 6 mm-diameter mice ear tissues and (**B**) the corresponding HE staining sections, CD45 positive cells counts (**C**) and immunohistochemical staining sections (**D**) under a 200X microscope. The results are represented as means ± SEM (*n* = 4–6). * *p* < 0.05, ** *p* < 0.01, **** *p* < 0.0001, compared with only the TPA treatment group.

A TPA-induced mouse ear edema inflammatory model was used to evaluate the in vivo anti-inflammatory effects of S2, S4 and S5 in the present study. This animal model is a reliable in vivo model system that mimics inflammatory skin diseases such as psoriasis and can therefore be used to assess the acute and chronic effects of drugs in skin inflammation [42]. Six hours after TPA application, the mean ear weight of mice increased from 7.6 mg to 23.4 mg, while the increase in mean ear weight in the dexamethasone, S2, S4 or S5 treatment groups was smaller than that in the TPA group. Compared with the TPA group, the mean ear weight of the S2, S4, or S5 treatment groups was 16.4 mg, 18.0 mg and 17.2 mg, respectively, which all showed significant inhibition on TPA-induced ear edema (Figure 6A). In addition, obvious histological lesions, including dermal edema and extensive inflammatory cell infiltration, was observed in the TPA-treated group. Treatment with S2, S4 and S5 effectively improved these pathological indicators (Figure 6B). CD45, also known as a leukocyte common antigen (LCA), is a marker of immune cells [43]. Visibly, the number of CD45 positive immune cells in a mouse ear was significantly increased by approximately 10-fold after TPA application, whereas dexamethasone, S2, S4, and S5 treatments resulted in a significant reduction in CD45 positive immune cells infiltration at the site of inflammation (Figure 6C,D).

### 3.8. EOZS Suppressed MAPK and NF-κB Pathways in TPA-Induced Mouse Model

**Figure 7:**
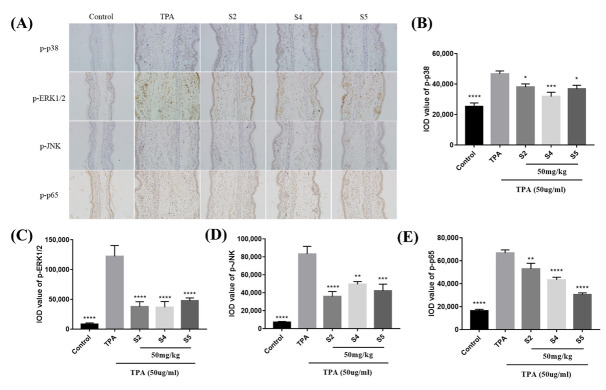
Effects of EOZS on MAPK and NF-κB signaling pathway-related proteins in TPA-induced mice ear tissues: (**A**) immunohistochemical staining sections under a 200X microscope and corresponding integrated optical density (IOD) values of (**B**) p-p38, (**C**) p-ERK1/2, (**D**) p-JNK and (**E**) p-p65. Data are shown as SEM (*n* = 3). * *p* < 0.05, ** *p* < 0.01, *** *p* < 0.001, **** *p* < 0.0001, compared with only the TPA treatment group.

We then attempted to investigate the effects of S2, S4 and S5 on MAPK and NF-κB signaling pathways in vivo. Immunohistochemical staining was performed to detect the expression levels of phosphor-p38, phosphor-ERK1/2, phosphor-JNK and phosphor-p65. The results indicated that TPA-induced mice ears showed a significant increase in the phosphorylation levels of p38, ERK1/2, JNK and p65 relative to unexposed ears. However, the S2, S4 and S5 treatment groups all reduced the expressions of these phosphorylated proteins to different degrees (Figure 7A–E). The above results demonstrated that S2, S4 and S5 could reduce inflammatory response in mouse ear by inhibiting MAPK and NF-κB signaling pathways, which is similar to the results in vitro.

## 4. Discussion

Natural products are used as traditional medicines for the treatment or prevention of a wide variety of human diseases due to their extensive pharmacological properties. As a kind of relatively safe natural product, plant-derived essential oils are more easily accepted by consumers [44]. To reduce substance abuse, the use of essential oils to relieve or treat diseases has also been developed as an alternative therapy [45]. *Z. striolatum* has traditional medicinal value, but the anti-inflammatory effect of its essential oil has not yet been reported. In the present work, we mainly analyzed the composition of EOZS from seven regions and evaluated their suppressive effect on inflammatory response and oxidant stress. Sixteen common chemical compounds were identified from the EOZS of seven regions, suggesting that these characteristic peaks may be used as the index components for quality control of *Z. striolatum*. From the phytochemical classification, *Z. striolatum* from seven locations could be divided into three clusters. The factors causing these differences may be related to the geographical location, climatic conditions and soil environment. Although the main chemical components and relative contents in S1–S5 were similar, the cytotoxicity of S1 was stronger than other samples. This indicated that it may be caused by minor or trace components in the S1.

Our research showed that EOZS from seven locations exhibited a certain degree of DPPH scavenging activity. Furthermore, they also significantly inhibited the production of NO and ROS in LPS-induced RAW 264.7 cells. Interestingly, EOZS from Huaihua City (S2), Zhangjiajie City (S4) and Enshi City (S5) showed a stronger inhibitory effect on NO production in LPS-induced RAW 264.7 cells. From the results of PCA and HCA, it can be seen that the main difference between cluster III (S1–S5) and other clusters included β-phellandrene, β-pinene, α-pinene and α-humulene. Lee et al. identified β-phellandrene as the main component of *Zanthoxylum schinifolium* essential oil, and found that *Zanthoxylum schinifolium* essential oil significantly inhibited the mRNA transcription level of inducible nitric oxide synthase [46]. There was direct evidence that α-pinene and α-humulene suppressed NO produced by activated macrophages or monocytes [47,48]. In addition, as the main component of the essential oil of *Xylopia parviflora*, β-pinene has been shown to reduce the NO production in LPS-induced RAW 264.7 cells [49]. The above evidence may partly explain the anti-inflammatory activity of S2, S4 and S5. S1 was not compared with other samples in cluster III at the same concentration because of its stronger cytotoxicity. However, the main components of S3 and S2, S4 and S5 were similar, whilst the effect of S3 was weaker, indicating that there may be an antagonistic effect between different components in S3.

Transcriptomics is a method to study the expression of RNA and the regulation of transcription in cells [50]. In recent years, transcriptomics has been widely used to reveal differences in the expression of genes in biological functions and to discover the mechanisms or targets of drugs. By using transcriptomics analysis, Li et al. found that Rebaudioside A could protect *Caenorhabditis elegans* from oxidative stress via regulating TOR and PI3K/Akt pathways [51]. RNA sequencing results of this study indicated that the MAPK and NF-κB pathways may be involved in the potential anti-inflammatory mechanism of EOZS. The MAPK signaling pathway plays an important role in extracellular signaling to the inner nucleus, and it can be activated by external environmental stress or stimulation by inflammatory mediators [52]. It has been shown that the conditional knockdown of the p38α MAPK gene results in a significant reduction in TNF-α production by LPS-induced microglia in mice, thus preventing the neuronal damage produced by LPS induction [53]. NF-κB, as a core regulator of inflammatory response, has been a popular research target in the field of inflammation and immunity. When canonical NF-κB signaling pathway is activated, IκB is phosphorylated via the IκB kinase (IKK) complex and subsequently polyubiquitinated and degraded by the 26S proteasome. NF-κB is then phosphorylated and enters the nucleus, leading to the initiation and protein expression of pro-inflammatory genes such as TNF-α and IL-6 [54]. All of this evidence suggests that the activation status of MAPK and NF-κB signaling pathways has a critical impact on the production of downstream pro-inflammatory mediators. Therefore, the inhibition of the excessive activation of MAPK and NF-κB signaling pathways is a strategy for the treatment of inflammation-related diseases.

**Figure 8:**
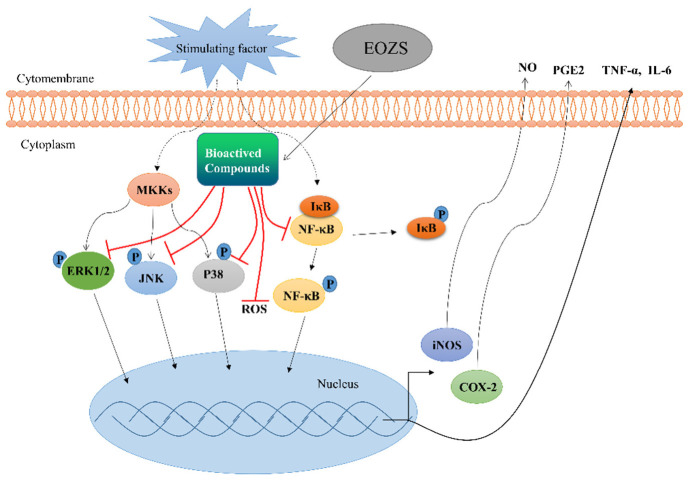
Schema showed the anti-inflammatory molecular mechanism of EOZS by activating MAPK and NF-κB signaling pathways.

During inflammation, cells damaged by irritation recruit immune cells to migrate towards the site of inflammation by releasing cytokines and chemokines, while immune cells produce more inflammatory mediators through activated MAPK and NF-κB signaling pathways [55], creating a positive feedback loop that may further exacerbate the progression of inflammation. In this study, we demonstrated that EOZS inhibited the activation of macrophage and production of pro-inflammatory mediators through the regulation of MAPK and NF-κB signaling pathways (Figure 8). In vivo experiments also showed that EOZS could reduce the infiltration of immune cells to the site of inflammation to alleviate inflammation. Indeed, ample evidence suggests that plant volatile oils possess anti-inflammatory pharmacological activity in vivo and in vitro [56,57,58].

## 5. Conclusions

In conclusion, this was the first study to clarify the anti-inflammatory molecular mechanism of EOZS, and these findings indicate that EOZS may have the potential to prevent or treat inflammation-related diseases. However, there are differences in the anti-inflammatory effects of EOZS from different locations. Therefore, it is necessary to establish relevant standards to control the quality of EOZS in order to promote its industrialization.

## Figures and Tables

**Table 1:** Chemical compounds of essential oils of *Z. striolatum* from different locations.

No	Compounds ^1^	Cal. RI ^2^	Lit. RI ^3^	Ref.	Relative Percentage (%)						
					S1	S2	S3	S4	S5	S6	S7
1	α-Thujene	920	924	[22]	0.28	0.23	0.17	0.24	0.24	-	0.04
2	α-Pinene	924	932	[22]	4.16	3.93	6.26	4.35	5.21	0.10	1.18
3	Camphene	941	946	[22]	0.15	0.11	0.21	0.14	0.24	-	0.03
4	β-Pinene	961	974	[22]	5.20	4.97	9.53	4.53	10.01	0.46	2.24
5	β-Myrcene	979	988	[22]	2.80	2.54	3.02	2.18	3.15	0.15	0.63
6	α-Phellandrene	994	1002	[22]	3.69	3.00	2.05	1.72	2.43	0.11	0.73
7	3-Carene	1000	1008	[22]	0.21	0.18	0.16	0.14	0.17	-	0.03
8	α-Terpinene	1004	1014	[22]	0.35	0.12	-	0.05	0.19	-	0.04
9	β-Phellandrene	1015	1025	[22]	23.34	22.27	20.57	18.38	21.27	8.81	5.60
10	(Z)-β-Ocimene	1029	1032	[22]	0.25	0.39	0.09	0.23	0.59	-	0.18
11	(E)-β-Ocimene	1037	1044	[22]	0.61	0.52	0.99	-	2.40	0.04	0.52
12	γ-Terpinene	1044	1054	[22]	2.31	1.48	1.28	0.39	2.10	0.09	0.12
13	Terpinolene	1080	1086	[22]	0.29	0.14	0.32	0.13	0.35	-	0.11
14	Linalool	1095	1095	[22]	-	-	0.53	-	0.51	-	0.09
15	Limonene oxide	1106	1132	[22]	-	-	-	-	0.15	0.05	-
16	(−)-endo-Fenchol	1109	1114	[22]	-	-	0.06	-	0.11	-	-
17	cis-p-Menth-2-en-1-ol	1116	1118	[22]	0.44	0.31	0.73	0.67	0.73	0.80	0.29
18	trans-p-Menth-2-en-1-ol	1137	1140	[22]	0.37	0.36	0.56	0.52	0.69	0.66	0.26
19	Pinocarvone	1154	1160	[22]	-	-	0.15	0.11	0.31	-	0.05
20	β-Pinene oxide	1164	1154	[22]	-	0.50	0.33	0.05	0.47	-	-
21	(−)-Terpinen-4-ol	1170	1174	[22]	0.82	0.26	1.10	0.80	1.06	1.83	0.43
22	Cryptone	1176	1183	[22]	3.08	5.74	4.21	5.34	4.47	7.57	1.13
23	α-Terpineol	1185	1186	[22]	-	-	-	-	-	-	0.43
24	(−)-Myrtenal	1187	1195	[22]	-	-	-	-	0.70	-	-
25	cis-Piperitol	1192	1195	[22]	0.33	-	1.33	0.27	0.99	0.44	-
26	(−)-Myrtenol	1201	1194	[22]	-	-	-	-	0.17	-	-
27	trans-Piperitol	1206	1207	[22]	0.28	0.24	0.13	-	-	-	0.07
28	2-Pentylcyclopentanone	1210	1208	[23]	-	-	0.27	0.20	0.26	0.36	-
29	Lilac alcohol D	1231	1232	[24]	-	-	-	0.07	-	0.19	-
30	Cumin aldehyde	1240	1238	[22]	-	-	0.18	0.31	0.23	0.47	0.05
31	Carvone	1244	1239	[22]	0.14	0.37	-	0.10	-	-	-
32	Piperitone	1255	1249	[22]	0.18	0.21	-	0.19	0.09	0.08	-
33	Phellandral	1264	1273	[22]	0.86	0.77	0.11	-	-	0.22	0.34
34	p-Menth-1-en-7-al	1267	1273	[22]	-	-	1.05	2.15	1.35	0.48	0.95
35	Isobornyl acetate	1279	1283	[22]	0.23	-	0.13	0.09	0.27	0.08	0.05
36	2-Caren-10-al	1284	1289	[25]	-	-	0.12	0.08	-	-	-
37	2-Undecanone	1291	1293	[22]	0.29	0.20	-	-	-	-	-
38	δ-Elemene	1327	1335	[22]	-	-	0.10	-	0.21	-	0.10
39	α-Cubebene	1337	1345	[22]	0.11	-	0.14	0.09	0.08	0.07	0.08
40	α-Copaene	1363	1374	[22]	0.34	0.29	0.35	0.39	0.26	0.36	-
41	β-cubebene	1376	1387	[22]	-	0.59	-	0.27	0.46	0.16	0.66
42	β-Elemene	1384	1389	[22]	2.99	3.01	3.76	2.12	3.33	2.21	4.97
43	α-Cedrene	1395	1410	[22]	-	-	-	0.42	-	-	0.11
44	α-Gurjunene	1396	1409	[22]	0.72	0.30	0.26	-	0.21	0.44	-
45	β-Caryophyllene	1407	1417	[22]	3.60	3.60	3.11	3.76	2.19	2.45	2.50
46	β-Humulene	1412	1436	[22]	-	-	0.08	-	-	-	0.28
47	γ-Elemene	1421	1434	[22]	0.07	-	0.27	-	-	-	0.51
48	α-Bergamotene	1425	1432	[22]	-	-	-	-	0.16	-	-
49	α-guaiene	1427	1437	[22]	-	-	-	-	-	-	0.44
50	Tetradecane	1430	1400	[22]	-	-	-	-	-	-	0.45
51	α-Humulene	1442	1452	[22]	12.38	12.19	12.36	12.66	8.77	11.58	5.03
52	Aromadendrene	1447	1441	[22]	-	-	-	-	-	-	0.16
53	(E)-β-Farnesene	1454	1454	[22]	-	-	0.16	-	0.08	-	-
54	allo-Aromadendrene	1467	1458	[22]	-	0.77	0.36	1.08	0.66	0.36	0.88
55	Germacrene D	1473	1484	[22]	-	-	0.23	-	0.17	2.40	1.46
56	Pentadecane	1478	1500	[22]	-	0.86	-	-	1.75	-	0.09
57	β-selinene	1482	1489	[22]	1.29	1.01	1.09	0.99	-	1.22	1.00
58	Valencene	1488	1496	[22]	-	-	0.95	-	0.46	-	0.82
59	Elixene	1490	1446	[26]	0.29	0.25	-	-	0.11	0.24	1.32
60	2-Tridecanone	1492	1495	[22]	-	-	-	0.14	-	-	-
61	(Z,E)-α-Farnesene	1493	1500	[27]	-	-	0.15	-	-	-	-
62	α-Selinene	1498	1498	[22]	0.44	0.47	-	0.40	0.40	0.23	0.39
63	β-Bisabolene	1500	1505	[22]	-	-	-	-	0.52	-	-
64	(E,E)-α-Farnesene	1503	1505	[22]	-	-	0.25	-	0.21	-	-
65	α-Panasinsen	1513	1518	[26]	0.16	0.15	-	0.14	-	0.14	0.07
66	δ-Cadinene	1517	1522	[22]	0.33	-	0.83	0.35	0.38	0.18	0.54
67	(Z)-Nerolidol	1540	1531	[22]	0.14	-	-	-	-	0.04	0.15
68	Germacrene B	1557	1559	[22]	-	-	0.80	0.39	0.63	0.14	3.61
69	Epiglobulol	1562	1532	[26]	-	-	-	-	-	0.09	-
70	(E)-Nerolidol	1567	1561	[22]	0.09	-	-	-	-	0.07	0.49
71	Caryophyllene oxide	1582	1582	[22]	1.58	1.42	1.35	2.86	1.30	3.33	3.12
72	Viridiflorol	1591	1592	[22]	-	-	-	-	0.13	0.28	-
73	Globulol	1595	1590	[22]	-	-	-	-	-	-	0.27
74	Tetradecanal	1603	1612	[22]	-	-	0.33	-	0.26	0.79	0.09
75	2,5,9-Trimethylcycloundeca-4,8-dienone	1609	-	-	3.31	3.61	3.64	7.12	2.95	8.09	1.80
76	selina-6-en-4-ol	1622	1624	[26]	-	-	0.10	0.12	-	-	0.36
77	Diethyl phthalate	1625	1639	[28]	-	-	-	-	-	0.24	-
78	Cubenol	1645	1645	[22]	-	-	0.25	0.46	0.10	0.18	1.71
79	1-Tetradecanol	1662	1671	[22]	-	0.60	-	0.84	0.10	0.59	3.46
80	α-Cadinol	1668	1652	[22]	1.58	0.96	2.26	1.15	1.49	1.01	0.65
81	β-Bisabolol	1676	1674	[22]	-	-	-	0.42	0.23	0.43	0.99
82	α-Eudesmol	1678	1652	[22]	-	-	-	-	-	2.96	-
83	2-Hexyl-1-decanol	1679	1673	[29]	-	-	-	1.72	-	-	-
84	Heptadecane	1680	1700	[22]	2.03	2.16	-	-	-	-	-
85	α-Bisabolol	1694	1685	[22]	-	-	-	-	-	0.49	-
86	2-pentadecanone	1698	1697	[22]	-	-	-	0.06	-	0.08	-
87	Pentadecanal	1710	1714	[30]	-	0.22	0.22	0.36	0.79	0.29	0.97
88	6-Isopropenyl-4,8a-dimethyl-1,2,3,5,6,7,8,8a-octahydro-naphthalen-2-ol	1714	1714	[31]	-	0.12	0.22	0.39	-	-	0.29
89	7R,8R-8-Hydroxy-4-isopropylidene-7-methylbicyclo[5.3.1]undec-1-ene	1725	1726	[32]	-	-	-	-	0.20	-	0.67
90	Isolongifolol	1755	1729	[22]	1.45	2.08	2.68	2.55	2.03	2.56	1.18
91	1-Octadecene	1777	1789	[22]	-	-	-	-	-	1.64	-
92	(Z)-7-hexadecenal	1790	1802	[33]	-	-	-	0.07	-	0.25	0.68
93	Hexadecanal	1813	1812	[27]	-	-	-	-	-	-	0.21
94	6,10,14-Trimethyl-2-pentadecanone	1844	1846	[30]	-	-	-	-	-	-	0.04
95	E-11(12-Cyclopropyl)dodecen-1-ol acetate	1876	-	-	-	-	-	-	-	-	0.26
96	Nonadecane	1887	1900	[22]	0.25	0.24	-	-	-	0.29	0.45
97	Linolenyl alcohol	1897	1901	[34]	-	0.19	-	-	-	-	-
98	(E,E)-farnesyl acetate	1928	1919	[30]	-	-	-	0.33	-	0.32	0.14
99	(Z,Z)-Geranyl linalool	1958	1960	[22]	-	-	-	-	-	0.07	0.10
100	α-Springene	1974	1986	[35]	-	-	-	-	-	0.08	0.19
101	Biformene	1975	1987	[36]	-	-	-	0.09	0.07	0.11	-
102	1-Heptadecanol	1977	1986	[37]	-	-	-	-	-	0.14	-
103	Eicosane	1982	2000	[30]	0.06	-	-	-	-	0.11	0.08
104	Dibutyl phthalate	1993	1962	[31]	-	-	-	-	-	-	0.43
105	Heptadecanoic acid	2022	2045	[36]	-	-	-	0.50	0.45	-	6.51
106	(E,E)-Geranyl linalool	2031	2026	[22]	-	-	-	-	-	-	0.58
107	Henicosane	2088	2100	[22]	0.48	0.38	0.10	0.14	0.22	0.69	0.50
108	13-Epimanool	2099	2036	[36]	-	-	-	-	0.11	-	0.14
109	Phytol	2135	2156	[32]	-	-	-	-	-	-	0.11
110	Docosane	2197	2200	[22]	-	0.26	0.12	-	-	2.04	0.23
111	Octadecanol acetate	2250	2209	[22]	-	-	-	-	-	0.19	0.27
112	cis-9-Tricosene	2289	2298	[27]	-	-	-	0.08	0.19	-	0.03
113	Tricosane	2331	2300	[22]	-	-	-	-	2.48	-	-
114	Tetracosane	2420	2400	[22]	4.25	4.01	1.15	1.38	-	6.94	3.55
115	Trans-squalene	2806	2810	[36]	-	-	-	0.64	-	-	-
116	1-Hexacosanol	2840	2852	[30]	-	-	-	-	-	2.49	-
	Total (%)				88.60	88.58	93.31	87.91	95.05	82.05	71.76

^1^ Identified compounds based on the GC–MS and RI analysis. ^2^ Retention indices (RIs) relative to n-alkanes (C_8_–C_40_) on the same silica capillary column. ^3^ Retention indexes (RIs) in the literature.

## Data Availability

Data is contained within the article and supplementary material.

## Supplementary Materials

The following are available online at https://www.mdpi.com/article/10.3390/antiox10122019/s1, Table S1: Geographic information of Z. striolatum collected from seven different habitats. Figure S1: Analysis of RNA-seq data in Ctrl, LPS and S4 groups. (A) Heatmap showed pearson correlation coefficient between each sample. (B) GO enrichment analysis of 45 DEGs upregulated by LPS and downregulated by S4. (C,D) Part of IL-17 signaling pathway and TNF signaling pathway obtained from Kyoto Encyclopedia of Genes and Genomes (KEGG, https://www.kegg.jp/, accessed on 21 September 2021).
