# Supplementary material for: Essential Oils from Zingiber striolatum Diels Attenuate Inflammatory Response and Oxidative Stress through Regulation of MAPK and NF-κB Signaling Pathways
Source: Antioxidants (Basel). 2021 Dec 19;10(12):2019. doi: 10.3390/antiox10122019 (PMC8698606; doi:10.3390/antiox10122019)
Supplement: Supplementary file 1 [file antioxidants-10-02019-s001.zip › antioxidants-1479209-supplementary.pdf]

# Supplementary Materials

## Tables and Figures

**Table S1.** Geographic information of *Z. striolatum* collected from seven different habitats

| Sample NO | Province | City | Locality | Collection Time | Storage Location |
|---|---|---|---|---|---|
| S1 | Sichuang | Yaan | Mingshan | 2018.09 | School of Biomedical and Pharmatheutical Sciences, Guangdong University of Technology |
| S2 | Hunan | Huaihua | Yuanling | 2018.09 | |
| S3 | Jiangsu | Nantong | Tongzhou | 2018.10 | |
| S4 | Hunan | Zhangjiajie | Sangzhi | 2018.10 | |
| S5 | Hubei | Enshi | Tujia and Miao Autonomous Prefecture | 2018.10 | |
| S6 | Hubei | Shiyan | Yunxi | 2018.09 | |
| S7 | Anhui | Liuan | Dabieshan | 2018.09 | |

(A)

(B)

(C)

(D)

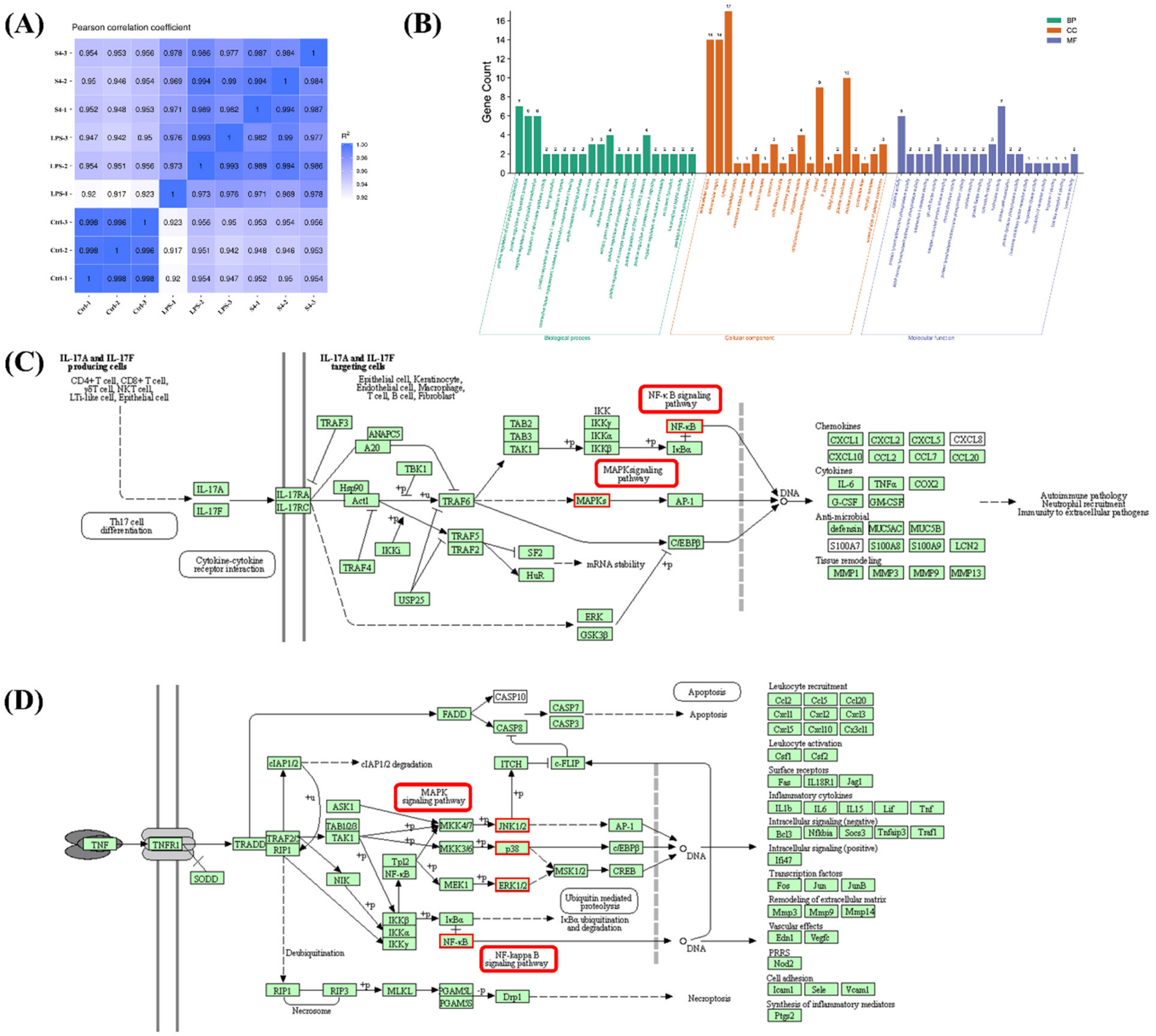

**Figure S1.** Analysis of RNA-seq data in Ctrl, LPS and S4 groups. (**A**) Heatmap showed pearson correlation coefficient between each sample. (**B**) GO enrichment analysis of 45 DEGs upregulated by LPS and downregulated by S4. (**C**,**D**) Part of IL-17 signaling pathway and TNF signaling pathway obtained from Kyoto Encyclopedia of Genes and Genomes (KEGG, https://www.kegg.jp/, accessed on 21 September 2021).
